# A COMPARATIVE STUDY OF GRIEF SUPPORT AND BURNOUT AMONG NURSING HOME STAFF

**DOI:** 10.1093/geroni/igad104.1076

**Published:** 2023-12-21

**Authors:** Frances Hawes, Shuangshuang Wang

**Affiliations:** University of Wisconsin-Eau Claire, Eau Claire, Wisconsin, United States; Southwest Jiaotong University, Chengdu, Jiangsu, China (People’s Republic)

## Abstract

A half-million older adults die in U.S. nursing homes (NHs) each year. However, only a few studies have been conducted on the experiences of the nursing home (NH) staff who provide this care. This study collected data from 555 NH workers in Fall 2022 and examined how their experiences with grief support affected burnout. Grief support was measured by the Grief Support Health Care Scale. Burnout was measured by the Maslach Burnout Inventory, which includes 3 subscales: exhaustion, depersonalization, and personal achievement. Findings indicate that 95% of sampled workers were exposed to at least one death at work; yet 31% percent of the sample never received any formal grief training. Examining the comparative dimensions of burnout, the highest percentage of workers with exhaustion and depersonalization ratings were RNs, nurse practitioners, and physicians, followed by CNAs. The lowest ratings of personal accomplishment were by direct support workers (maids/janitors housekeeping and laundry food service workers). Controlling for NH and individual characteristics, multivariate logistic regression showed that compared to workers who received low grief support, those who received high grief support were more likely to report high personal achievement and low feelings of depersonalization (i.e., impersonal response to residents). This research provides valuable information about the importance of grief support for nursing home workers to reduce feelings of burnout. In addition, findings indicate that there is a difference in grief support and burnout between different categories of nursing staff and there is a need to provide increased grief support to frontline workers.

